# Endoscope‐Assisted Transcervical Resection of Parapharyngeal Space Tumors

**DOI:** 10.1002/ohn.976

**Published:** 2024-10-01

**Authors:** Joshua D. Smith, Steven B. Chinn, Shaum Sridharan, Kevin J. Contrera, Molly E. Heft‐Neal, Matthew E. Spector

**Affiliations:** ^1^ Department of Otolaryngology–Head and Neck Surgery University of Pittsburgh Medical Center Pittsburgh Pennsylvania USA; ^2^ Department of Otolaryngology–Head and Neck Surgery University of Michigan Ann Arbor Michigan USA

**Keywords:** endoscope, endoscopic, parapharyngeal space, transcervical, tumors

## Abstract

**Objective:**

We describe a novel technique for endoscope‐assisted (EA) transcervical (TC) approach for resection of parapharyngeal space (PPS) tumors and compare perioperative outcomes of this approach to standard TC approaches.

**Study Design:**

Retrospective chart review.

**Setting:**

Single tertiary care center.

**Methods:**

This was a single‐institution, retrospective analysis of all patients undergoing TC approach for resection of PPS tumors over a 10‐year period. We describe unique advantages of our surgical approach utilizing a 0° endoscope for improved surgical access, visualization, and efficiency. *χ* ^2^ and Student's *t* test were used to compare perioperative outcomes between cases in which an endoscope was utilized EA for resection versus standard TC approach.

**Results:**

Our cohort included 77 patients (n = 40 EA, n = 37 TC). There was no difference in patient age, sex, tumor laterality, tumor size, or tumor location between groups. The EA approach was associated with significantly shorter operative times (median [range] for EA 73 [33‐270] minutes vs TC 112 [56‐362] minutes, *P* < .01) and reduced rates of immediate postoperative marginal mandibular nerve paresis (EA: n = 5 [12.5%] vs TC: n = 16 [43.2%], *P* < 0.01).

**Conclusion:**

EA TC approach for resection of PPS tumors offers improved surgical access and is associated with reduced surgical time and rates of marginal mandibular nerve paresis compared to standard transcervical approaches.

Tumors of the parapharyngeal space (PPS) are rare, accounting for <1% of all neoplasms of the head and neck.^1^ As most (>80%) PPS tumors are benign lesions of salivary or neurogenic origin, surgical excision remains the mainstay of treatment, with radiation and/or chemotherapy reserved for aggressive malignancies.^2^ Excision of PPS tumors poses unique challenges, owing to narrow corridors for surgical access, intricate neurovascular anatomy, and potential for significant surgical morbidity.^3^

The optimal surgical approach for PPS tumor resection is guided by complex factors, including tumor histology (eg, benign or malignant), size, anatomic compartment (ie, pre‐ vs poststyloid), proximity to the skull base, and surgeon comfort.^4^ Numerous approaches, each with unique advantages and limitations, have been described, including transcervical (TC), TC‐transparotid, mandibular split, transoral, and transnasal.^2^, ^5^, ^6^ In particular, the TC approach affords shorter operative times, avoids extensive facial nerve dissection, and permits direct exposure for access to most PPS tumors. Noted limitations of this approach include suboptimal visualization of the superior PPS and narrow surgical corridor.^7^

In recent years, endoscope‐assisted (EA) transoral approaches to the PPS have been increasingly applied in select patients.^8^, ^9^ Endoscopes facilitate a widely magnified operative view, versatility of surgical instrumentation and dexterity, and improved visualization of the internal carotid artery (ICA), internal jugular vein (IJV) and cranial nerves (CNs) IX to XII in the poststyloid PPS.^10^ Further, endoscopes may be associated with reduced blood loss and rates of postoperative facial nerve paresis compared to standard transoral approaches.^8^

EA TC approach to the PPS has been described in cadaveric studies and limited case series, but its unique advantages and associated outcomes for patients undergoing excision of PPS tumors have yet to be established.^11^ Here, we describe our surgical technique for EA TC resection of PPS tumors and compare perioperative outcomes of this approach to standard TC approaches.

## Methods

This was a single‐institution, retrospective cohort study of all patients undergoing TC approach for resection of PPS tumors over a 10‐year period (2013‐2023). Inclusion criteria stipulated age ≥18 years and tumors primarily of the PPS with limited extension into the deep lobe of the parotid gland (defined as medial to the retromandibular vein). Patients with both benign and malignant PPS tumors were included. This study was approved by the University of Michigan Institutional Review Board (HUM00050982).

**Figure 1:**
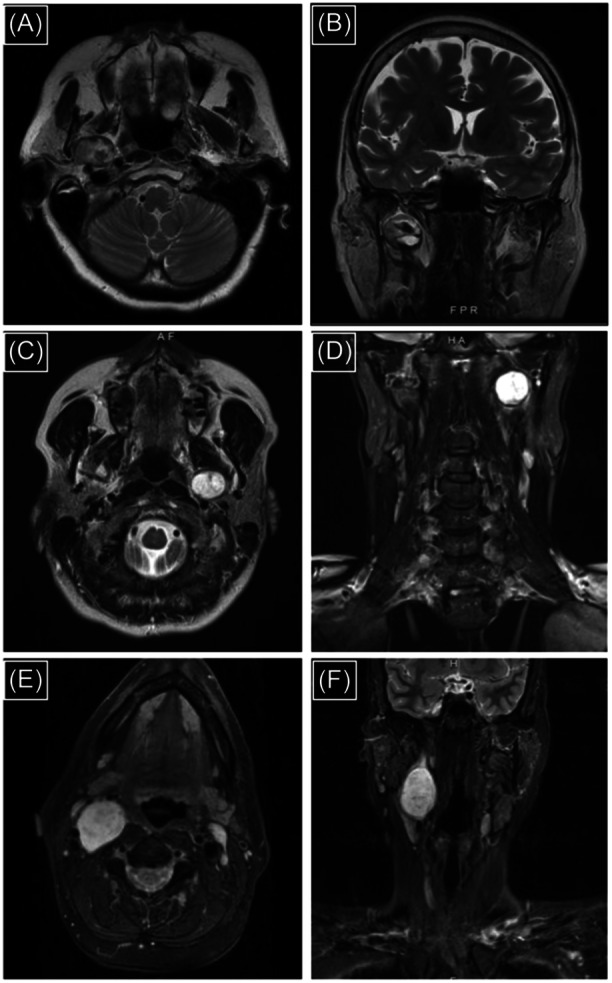
Use of endoscopes for resection of parapharyngeal space tumors optimizes surgical access, visualization, and efficiency in diverse clinical scenarios. Illustrative examples depicted here. 2.4 cm pleomorphic adenoma of the right prestyloid PPS (A, T2‐weighted axial MRI and B, T2‐weighted coronal MRI). 2.3 cm acinic cell carcinoma of the left prestyloid PPS adjacent to skull base (C, T2‐weighted axial MRI and D, T2‐weighted coronal MRI). 4.5 cm sympathetic chain schwannoma of the right poststyloid PPS (E, T2‐weighted axial MRI and F, T2‐weighted coronal MRI). All were resected without complication using an endoscope‐assisted approach. MRI, magnetic resonance imaging; PPS, parapharyngeal space.

Patients in our study underwent either EA or standard TC approach for excision of PPS tumors based on surgeon preference (Figure 1). In all patients, an incision was made approximately 2 cm inferior to the mandible, the submandibular gland was translocated anteriorly, and the posterior belly of the digastric was identified and skeletonized toward the mastoid tip. The medial border of the sternocleidomastoid was then dissected to expose the ICA and IJV in the superior neck. The sternocleidomastoid, submandibular gland, and mandible were retracted and the stylomandibular ligament was sharply released to fully expose the PPS.

**Figure 2:**
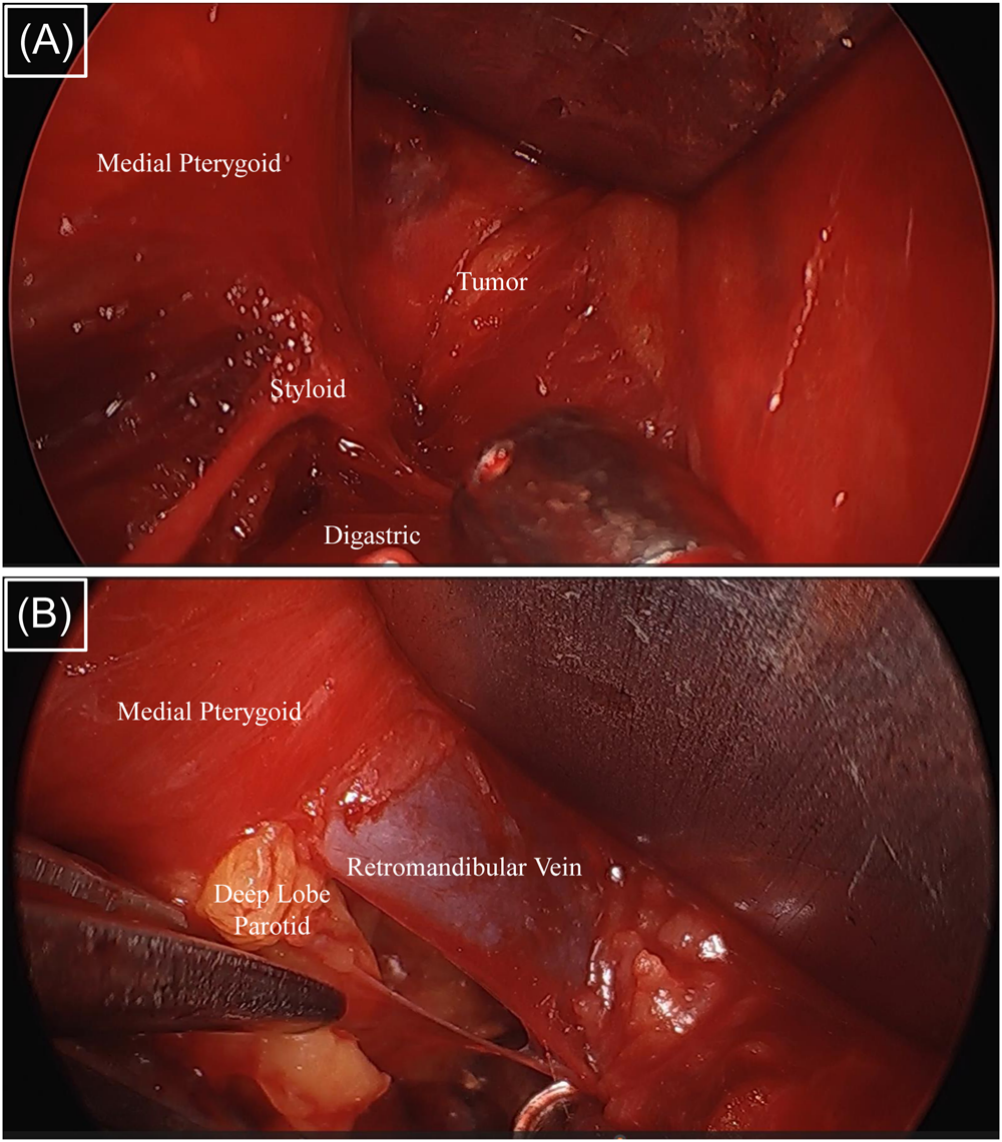
(A, B) Representative intraoperative photos of the endoscope‐assisted approach showing high‐resolution visualization of tumor‐normal tissue interfaces.

Tumor dissection then proceeded, and in the EA group only, a 0° endoscope (Karl Storz SE & Co KG) was used to improve visualization of tumor and adjacent neurovascular structures during tumor dissection (Figure 2). This dissection required 2 surgeons. The first surgeon held the endoscope and provided retraction, permitting the second surgeon bimanual dexterity for intricate tumor dissection. All tumors in the EA group were removed by EA TC approach only without identification or dissection of the facial nerve main trunk. After tumor excision, hemostasis was achieved, and the wound was closed in layers over a Jackson Pratt drain. All patients were planned for overnight hospital stay with drain removal and discharge on the first postoperative day.

From each patient chart, we collated the following variables: patient age, sex, tumor size, laterality, and location (ie, pre‐ or poststyloid), and surgical approach (ie, EA or TC). Intraoperative outcomes of interest included rate of tumor capsule violation, operative time, blood loss, and inadvertent injury to ICA, IJV, or CN IX to XII. Postoperative outcomes of interest included rate of any complication, rate of marginal mandibular nerve paresis, tumor histology, margin status, and disease recurrence.

*χ* ^2^ and Student's *t* test were utilized for comparison of categorical and continuous variables, respectively, between EA and TC resection groups. SPSS version 29 (IBM SPSS) was used for statistical analyses. Statistical significance was defined as *P* ≤ .05 for all tests.

## Results

Our analytic cohort included 77 patients. Forty (52%) underwent EA approach and 37 (48%) underwent TC approach for resection of PPS tumors. EA and TC groups were balanced for most demographic and tumor factors, although the EA group had a higher proportion of malignant tumors (Table 1).

**Table 1:** Comparison of Demographic and Tumor Factors in Patients Undergoing EA Versus TC Approach to Resection of PPS Tumors

	Overall cohort (n = 77)	EA group (n = 40)	TC group (n = 37)	*P* value
Age, y	54 (1‐76)	53 (13‐74)	55 (1‐76)	.48
Sex				
Male	29 (38%)	14 (35%)	15 (40%)	
Female	48 (62%)	26 (65%)	22 (60%)	.62
Tumor laterality				
Right	40 (52%)	21 (53%)	19 (51%)	
Left	37 (48%)	19 (47%)	18 (49%)	.92
Tumor size, cm	3.5 (1‐7.9)	3.4 (1‐6.8)	4 (1‐7.9)	.17
Tumor location				
Prestyloid	61 (79%)	30 (75%)	31 (84%)	
Poststyloid	16 (21%)	10 (25%)	6 (16%)	.34
Tumor histology				
Pleomorphic adenoma	45 (58%)	19 (48%)	25 (68%)	
Other benign tumors^a^	20 (26%)	14 (35%)	7 (19%)	
Malignant tumors^b^	12 (16%)	7 (17%)	5 (13%)	.04

Data presented as n (%) or median (range).

Abbreviations: EA, endoscope‐assisted; PPS, parapharyngeal space; TC, traditional transcervical.

^a^ Including schwannoma (n = 6), vascular malformation (n = 5), paraganglioma (n = 3), Warthin's tumor (n = 3), oncocytoma (n = 2), and benign lymph node (n = 1).

^b^ Including acinic cell carcinoma (n = 3), salivary ductal carcinoma (n = 2), papillary thyroid cancer (n = 1), rhabdomyosarcoma (n = 1), neuroblastoma (n = 1), malignant paraganglioma (n = 1), adenocarcinoma (n = 1), hyalinizing clear cell carcinoma (n = 1), and malignant solitary fibrous tumor (n = 1).

The EA approach was associated with significantly shorter operative times compared to the TC approach for all patients (median [range] of 73 [33‐270] vs 112 [56‐362] minutes, *P* = .008) and for benign tumors only (median [range] of 65 [33‐270] vs 112 [56‐362] minutes, *P* = .005). There was no difference in blood loss between groups overall (median [range] for EA: 20 [2‐1450] vs TC: 25 [5‐1600] cc, *P* = .59) and when considering only benign tumors (median [range] for EA: 20 [2‐300] vs TC: 25 [5‐1600] cc, *P* = .24).

When analyzing pleomorphic adenomas only, there was no difference in inadvertent capsule rupture intraoperatively between groups (EA: n = 2 [5%] vs TC: n = 4 [11%], *P* = .34). When analyzing malignant tumors only, there was no difference in positive margin rate between groups (EA: n = 1 [2.5%] vs TC: n = 2 [5.5%], *P* = .58). No inadvertent injuries to the ICA, IJV, or CN IX to XII were seen in either group.

Postoperative outcomes in EA and TC groups are shown in Table 2. Notably, the rate of documented marginal mandibular branch paresis postoperatively in the EA group was nearly one‐fourth that of the TC group (n = 5 [12%] vs n = 16 [43%], *P* = .003).

**Table 2:** Comparison of Postoperative Outcomes in Patients Undergoing EA Versus TC Approach to Resection of PPS Tumors

	Overall cohort (n = 77)	EA group (n = 40)	TC group (n = 37)	*P* value
Length hospital stay, d	1 (1‐21)	1 (1‐5)	1 (1‐21)	.23
Complication, marginal mandibular nerve paresis				
No	56 (73%)	35 (88%)	21 (57%)	
Yes	21 (27%)	5 (12%)	16 (43%)	.003
Complication, other^a^				
No	73 (95%)	38 (95%)	35 (95%)	
Yes	4 (5%)	2 (5%)	2 (5%)	.94
Tumor recurrence				
No	74 (96%)	39 (98%)	35 (95%)	
Yes	3 (4%)	1 (2%)	2 (5%)	.51

Data presented as n (%) or median (range).

Abbreviations: EA, endoscope‐assisted; PPS, parapharyngeal space; TC, traditional transcervical.

^a^ Including hematoma requiring reoperation (n = 3), and surgical site infection requiring antibiotics (n = 1).

## Discussion

Utilizing the largest published cohort of patients undergoing EA approach for resection of PPS tumors to date, we posited that the EA approach for resection of PPS tumors could provide improved surgical efficiency with reduced morbidity compared to the standard TC approach. EA approach was associated with a reduction in median operative time of approximately 40 minutes compared to the standard TC approach. Further, the rate of marginal mandibular nerve paresis after EA approach was approximately one‐fourth that seen with the TC approach (Table 2). Our findings illustrate the unique advantages of the EA technique for PPS tumors.

The standard TC approach to the PPS is limited by a narrow, deep working corridor for dissection and poor visualization of the superior PPS.^12^ Stable retraction of soft tissues overlying the mandible necessary for adequate exposure of the PPS may lead to traction or compression injury of the marginal mandibular nerve. Further, a narrow working corridor may limit surgical dexterity and instrumentation critical for meticulous tumor dissection.^13^ Due to poor visualization of the superior PPS, blind finger dissection is typically used to free tumor attachments superiorly, which may increase the risk of capsule rupture, bleeding, and injury to the ICA, IJV, or CN IX to XII.^14^ Alternative approaches, including TC‐transparotid and transmandibular routes, improve visualization and access to large and/or superior PPS tumors but are associated with considerable morbidity.^15^

The EA approach for resection of PPS tumors significantly mitigates each of these limitations. The versatility and utility of this approach were evident in diverse clinical scenarios, including cases of prestyloid PPS pleomorphic adenomas, malignant tumors, and large poststyloid PPS schwannomas (Figure 1). High‐resolution optics provided by endoscopes permit continuous visualization of tumor‐normal tissue interfaces around the entire tumor periphery, especially critically for lobulated and malignant tumors (Figure 2).^8^, ^16^ Further, endoscopic magnification anecdotally improves recognition of both small and large vasculature (eg, ICA, IJV) in the PPS and facilitates meticulous hemostasis during tumor dissection.^11^ Each of these factors contributed to significantly shorter operative times in the EA group in our study. With endoscopic assistance, less soft tissue retraction is necessary for adequate PPS exposure. This explains the significantly lower rate of marginal mandibular nerve paresis in the EA group in our study, which benefits such patients in the postoperative period.

The EA approach proved safe and effective for resection of all tumor sizes, both benign and malignant, and tumors in all anatomic compartments of the PPS, including for poststyloid tumors. Critical oncologic outcome metrics, including rate of capsule rupture (benign tumors), rate of positive margins (malignant tumors), and tumor recurrence, were low (all ≤5%) and nearly identical to the TC approach. Others have advised against utilizing minimally invasive endoscopic techniques for resection of malignant or hypervascular (eg, paraganglioma) tumors within the PPS.^16^ Here, we utilize a standard TC incision, 3 to 4 cm in length, and thus do not classify our technique as minimally invasive. This incision facilitates delivery of large PPS tumors through the neck, rather than via a transoral route utilizing TORS platforms, as described in Duek et al.^13^, ^14^

Limitations of our study include its single‐center, retrospective design. Further, variability in individual surgeon approach and preferences may have contributed to differences in outcomes. In our cohort, 3 surgeons operated in the TC group and 2 surgeons operated in the EA group. We could not control for any potential bias in this regard. While ours is the largest published study of EA TC approach for resection of PPS tumors, our relatively small sample may have limited detection of differences in blood loss or postoperative outcomes between the EA and TC approaches. Further, marginal mandibular branch paresis was determined by postoperative physical exam, prior to hospital discharge, by resident and/or attending physicians who were aware of surgical approach thus adding possible bias to our study. Our study provides evidence for superiority of the EA approach as it pertains to operative efficiency and postoperative marginal mandibular branch paresis. However, larger comparative studies are needed. Further, while we did not collect hospital cost data for this cohort, we surmise that significantly reduced operative times in the EA group directly translated to reduced expenditures. Future studies may help confirm this association.

## Conclusions

EA TC approach for resection of PPS tumors optimizes surgical access and was associated with reduced surgical time and rate of immediate postoperative marginal mandibular nerve paresis compared to TC approaches.

## Author Contributions

**Joshua D. Smith**, substantial contributions to conception or design of the work and acquisition, analysis, or interpretation of data, drafting the work and reviewing it critically for intellectual content, final approval of the version to be published, agreement to be accountable for all aspects of the work; **Steven B. Chinn**, substantial contributions to conception or design of the work and acquisition, analysis, or interpretation of data, drafting the work and reviewing it critically for intellectual content, final approval of the version to be published, agreement to be accountable for all aspects of the work; **Shaum Sridharan**, substantial contributions to conception or design of the work and acquisition, analysis, or interpretation of data, drafting the work and reviewing it critically for intellectual content, final approval of the version to be published, agreement to be accountable for all aspects of the work; **Kevin J. Contrera**, substantial contributions to conception or design of the work and acquisition, analysis, or interpretation of data, drafting the work and reviewing it critically for intellectual content, final approval of the version to be published, agreement to be accountable for all aspects of the work; **Molly E. Heft‐Neal**, substantial contributions to conception or design of the work and acquisition, analysis, or interpretation of data, drafting the work and reviewing it critically for intellectual content, final approval of the version to be published, agreement to be accountable for all aspects of the work; **Matthew E. Spector**, substantial contributions to conception or design of the work and acquisition, analysis, or interpretation of data, drafting the work and reviewing it critically for intellectual content, final approval of the version to be published, agreement to be accountable for all aspects of the work.

## Disclosures

### Competing interests

The authors report no potential conflicts of interest related to this work.

### Funding source

None.

## References

[1] López F , Suárez C , Vander Poorten V , et al. Contemporary management of primary parapharyngeal space tumors. Head Neck. 2019;41(2):522‐535. 30549361 10.1002/hed.25439

[2] Matsuki T , Okamoto I , Tada Y , et al. Resection of parapharyngeal space tumors located in the prestyloid compartment: efficacy of the cervical approach. Ann Surg Oncol. 2021;28:3066‐3072. 33141372 10.1245/s10434-020-09268-x

[3] Olsen KD . Tumors and surgery of the parapharyngeal space. Laryngoscope. 1994;104(5 pt 2 suppl 63):1‐28. 10.1288/00005537-199405000-00001 8189998

[4] Lombardi D , Ferrari M , Paderno A , et al. Selection of the surgical approach for lesions with parapharyngeal space involvement: a single‐center experience on 153 cases. Oral Oncol. 2020;109:104872. 32659725 10.1016/j.oraloncology.2020.104872

[5] Li L , Xu H , London Jr, NR , Carrau RL , Jin Y , Chen X . Endoscopic transoral approach to the lateral poststyloid space. Head Neck. 2023;45:294‐301. 36333984 10.1002/hed.27240

[6] Li L , Zhou J , Xu H , Jin Y , Chen X , Carrau RL . Maximal exposure of the parapharyngeal internal carotid artery via transnasal and transoral corridors. Head Neck. 2023;45(3):757‐763. 36513521 10.1002/hed.27267

[7] Kuet ML , Kasbekar AV , Masterson L , Jani P . Management of tumors arising from the parapharyngeal space: a systematic review of 1,293 cases reported over 25 years. Laryngoscope. 2015;125(6):1372‐1381. 25448637 10.1002/lary.25077

[8] Chen H , He Z , Li G , et al. Endoscopy‐assisted transoral approach to resect parapharyngeal space tumors: a systematic review and meta‐analysis. Laryngoscope. 2021;131(10):2246‐2253. 33616215 10.1002/lary.29458

[9] Larson AR , Ryan WR . Transoral excision of parapharyngeal space tumors. Otolaryngol Clin North Am. 2021;54(3):531‐541. 34024481 10.1016/j.otc.2021.03.001

[10] Tang R , Mao S , Liu S , Li Z , Zhu H , Zhang W . Types of endoscopic surgical approaches for benign parapharyngeal space tumors. Oral Oncol. 2022;130:105875. 35576887 10.1016/j.oraloncology.2022.105875

[11] Fang Y , Wu H , Tan AD , Cheng L . Transcervical endoscopic approach for parapharyngeal space: a cadaver study and clinical practice. Acta Otolaryngol. 2020;140:163‐169. 31933416 10.1080/00016489.2019.1663924

[12] Chang SS , Goldenberg D , Koch WM . Transcervical approach to benign parapharyngeal space tumors. Ann Otol Rhinol Laryngol. 2012;121(9):620‐624. 23012902 10.1177/000348941212100910

[13] Duek I , Amit M , Sviri GE , Gil Z . Combined endoscopic transcervical‐transoral robotic approach for resection of parapharyngeal space tumors. Head Neck. 2017;39:786‐790. 28139028 10.1002/hed.24685

[14] Duek I , Sviri G , Billan S , Gil Z . Minimally invasive surgery for resection of parapharyngeal space tumors. J Neurolog Surg B Skull Base. 2018;79:250‐256. 10.1055/s-0037-1607315 PMC5951708 29765822

[15] Benet A , Plata Bello J , El‐Sayed I . Combined endonasal‐transcervical approach to a metastatic parapharyngeal space papillary thyroid carcinoma. Cureus. 2015;7:e285. 26203403 10.7759/cureus.285 PMC4509622

[16] Pilolli F , Giordano L , Galli A , Bussi M . Tumori dello spazio parafaringeo: approccio transcervicale video‐assistito mini invasivo. Acta Otorhinolaryngol Ital. 2016;36:259‐264. 27734977 10.14639/0392-100X-709 PMC5066460

